# The physiological demands of intermittent endurance exercise performed at different intensities within the anaerobic power reserve: Implications for fatigue development and performance

**DOI:** 10.1371/journal.pone.0359318

**Published:** 2026-11-13

**Authors:** Joao Henrique Falk Neto, Kelvin E. Jones, Aidan K. Comeau, Normand Boulé, Michael D. Kennedy

**Affiliations:** 1 Faculty of Kinesiology, Sport, and Recreation, University of Alberta, Edmonton, Alberta, Canada; 2 Neuroscience and Mental Health Institute, Edmonton, Alberta, Canada; 3 Alberta Diabetes Institute, University of Alberta, Edmonton, Canada; University of Urbino: Universita degli Studi di Urbino Carlo Bo, ITALY

## Abstract

This study aimed to describe the physiological demands of intermittent endurance exercise (cycling) performed at different intensities within the anaerobic power reserve (APR); assess if these intensities are sustainable; and determine their effect on subsequent performance. On three separate days, endurance-trained males (n = 15, 36.2 ± 9.4 years old; 84.9 ± 13.0 kg) completed a high-intensity intermittent protocol consisting of fifteen 10-second high-intensity efforts (i.e., surges in intensity) separated by 50 seconds of low intensity (60% maximal aerobic power – MAP) work. These surges were performed either at the intensity associated with the participant’s MAP, or at 25% or 50% of their APR. A 30-second Wingate test (WG) was performed immediately after the protocol. Greater physiological and perceptual demands occurred during the APR conditions compared to MAP (p < .05), with very high demands occurring early in the protocol (surges 3–6) during the APR conditions. As such, only 8 and 4 participants completed all surges in the 25APR and 50APR conditions, respectively. Mean power output in the WG was significantly different between all conditions (p < .001), while maximal peak power (MPP) was only different between MAP and 50APR (p = .006). The results indicate that intermittent work at 25% and 50% APR lead to rapid increases in key physiological markers, altering the intramuscular environment, resulting in both severe leg discomfort and a perceived inability to cope with recurring surges. Consequently, the ability to perform a finishing sprint is also affected. Athletes involved in endurance events that require supramaximal efforts should consider the impact of performing high-intensity intermittent work relative to their ability to stay in the race and to maintain power output during the finishing sprint.

## Introduction

In endurance events with a variable pacing profile, the athletes’ work rate or exercise intensity alternates between periods of low (below the first ventilatory threshold – VT1) and high (above the maximal metabolic steady state – MMSS) intensity work [1]. These variations in intensity can be so frequent that these races have been termed “intermittent endurance events” [2]. In sports such as triathlon and mountain biking, for example, athletes perform an average of one surge of 6–15 seconds in duration per minute [2–4].

Many of these surges occur at supramaximal intensities (i.e., above the athlete’s maximal aerobic power – MAP, the maximal power output attained in a graded exercise test), ranging between 110–160% of the athlete’s MAP, depending on the length of each surge [2,3,5]. Efforts at even higher intensities have been reported in races, with surges representing 200–300% MAP at the start of mountain bike races [2,4,5], or approaching the athlete’s maximal peak power (MPP, the maximal power generated in 1-second during an all-out effort) in triathlon and road cycling [3,6].

These supramaximal efforts occur within the athlete’s anaerobic power reserve (APR), the range of intensities between the athlete’s MAP and their MPP [7–9]. Within the APR, exercise intensity and duration follow an exponential relationship, with fatigue developing quickly at higher intensities due to increased metabolic and neuromuscular demands [10]. Indeed, exercise within the APR requires a significant contribution from anaerobic energy sources, leading to high blood lactate levels, along with an increased recruitment of type II muscle fibers and greater electromyographic activity (EMG), signaling an increase in motor unit activity [11–13]. Work at these intensities leads to partial depletion of phosphocreatine (PCr) stores, accumulation of fatigue-related metabolites, and a reduction in muscle pH [12^–^14], with these factors contributing to the significant development of peripheral fatigue. Consequently, the athlete’s ability to sustain exercise at these intensities, and to perform a finishing sprint, crucial in many endurance events, is impaired [15].

Exercise tolerance at these intensities, therefore, is related to the percentage of the APR at which these efforts are performed. A lower intensity within the APR leads to less reliance on anaerobic energy production, and consequently, fewer metabolic disturbances that are linked to fatigue and that could hinder subsequent performance [7,16]. In intermittent endurance events, many of the efforts occur around 20% to 30% of the athlete’s APR 2,3, with the most intense surges occurring at approximately 50% of the athlete’s APR 2, an intensity that can only be sustained briefly during continuous or intermittent work [8,10,17,18]. However, in these events, the periods of low intensity work (e.g., 40–60 seconds, typically performed at 40–60% MAP) between efforts allow recovery and clearance of fatigue-related metabolites [2,4,11,12], and minimize the increases in muscle activity and oxygen consumption [11,12]. As such, this pattern can be sustained for long durations (e.g., 60–90 minutes in mountain biking and Olympic distance triathlon) [2,3].

Given that each athlete has a different locomotor profile (i.e., different values for MAP and MPP, and consequently, a different range of intensities for APR), a surge in intensity during a race means that each athlete would perform these efforts at different percentages within their APR. Therefore, understanding whether intermittent exercise within the APR is sustainable, and at which intensity level, can provide useful information for those involved in these events.

Thus, the aims of this study were to 1) describe the physiological responses and the changes in perceived and performance fatigability during an intermittent endurance protocol performed at two different intensities (25% and 50%) within the athlete’s APR, 2) to understand if these intensities are sustainable over 15 minutes of intermittent work, and 3) to determine the effect of this intermittent profile on the athlete’s ability to perform a finishing sprint. A third condition, with the efforts performed at the intensity associated with the athletes’ MAP, was performed to provide a comparison to a known sustainable intensity during intermittent efforts. It was hypothesized that both APR conditions would lead to very high levels of metabolic stress, proportional to the intensity of the efforts. However, the intermittent protocol would allow the participants sufficient time to recover between the high-intensity efforts, allowing each condition to be tolerable for 15 surges. Lastly, it was hypothesized that these demands would significantly impair the athlete’s ability to perform a finishing sprint, with performance decrements proportional to the intensity of each condition.

## Materials and methods

### Participants

The study is a randomized controlled trial that recruited 15 trained male endurance athletes. Participants were age group athletes, considered tier 2 endurance athletes [19], competing at a regional, national, or international level in cycling and triathlon, had 3 + years of training experience, and were currently training more than 4 hours per week. The sample size was chosen to match similar studies in the literature. All the participants were informed of the procedures of the study prior to providing written informed consent to participate. The study was approved by the University of Alberta Ethics Committee (Pro 00115158). Data was collected between 10/01/2022 and 20/07/2022.

### Procedures

Every participant completed all tests at the same time of the day (± 2 hours) and under similar temperature and humidity conditions (23.2 ± 0.2°C, 39.2 ± 12.4%). Each testing day was separated by at least 48 hours of rest. The participants were instructed to avoid strenuous exercise on the two days preceding the test, to maintain a similar sleeping routine prior to each testing day, avoid caffeine and alcohol in the 12 and 24 hours prior to the test, respectively, and to ensure that they consumed a similar light meal 2–4 hours before each test. All the participants verbally confirmed that they followed these instructions.

### Test protocols

Each participant visited the lab on 4 different occasions. All tests were performed on the same electronically braked cycling ergometer (Velotron Racermate, Seattle, WA). During the first day of testing, the participants were instructed to adjust the ergometer (seat, stack, and handlebar height) to their preference. This setup was recorded and replicated on each subsequent trial.

### Determination of maximal aerobic power (MAP), maximal peak power (MPP), and anaerobic power reserve (APR)

A graded exercise test was performed to determine the participants’ MAP. After a 6-minute warmup (100W), the test started with the participants cycling at an intensity of 125W, with the intensity increasing by 30W every 2 minutes until the attainment of volitional exhaustion or until the participants were unable to maintain a cadence higher than 60 rpm despite strong verbal encouragement. The participant’s MAP was calculated based on the formula below [18]:


$$\begin{array}{c} MAP=P+(t/120\times 30) \end{array}$$

Where *P* is the power output of the last completed stage (in watts) and *t* is the time completed in the last non-completed stage (in seconds).

The participant’s MPP was determined via a 6-second Wingate test [19], performed 15 minutes following the graded exercise test. The test was performed from a “flying” start with the participants asked to reach their maximal cadence at the end of a 5-second ramp-up period, prior to a load equivalent to 7.5% of the participants’ bodyweight being applied. The Velotron software records the highest power output attained during the test as the maximal peak power. The participants completed three 6-second Wingate tests, with 3 minutes of active rest between each test. The highest of the 3 MPP values was considered as the participant’s MPP. The test’s inter- and intra-day reliability is excellent (intraclass correlation coefficient ICC > 0.90) [20].

The anaerobic power reserve was calculated as the difference between the participant’s MPP and MAP [7,18,21].

### High intensity intermittent protocols

On visits 2–4, the participants completed one of three different repeated high-intensity intermittent protocols. Prior to the start of the test, the participants performed a 6-minute warm up pedalling at 110W, with short (6-second) accelerations against a load equivalent to 70%, 75%, and 80% of their maximal aerobic power (MAP) at 2, 4, and 6 minutes. After 1 minute of passive rest, the participants then pedalled at a low intensity (100W) for 1 minute, with the test starting immediately at the end of this 1-minute period.

The protocol consisted of fifteen 10-second high-intensity efforts (surges) separated by 50 seconds of low intensity (60% MAP) cycling. For all participants, this intensity was below the first ventilatory threshold (VT1), determined based on an increase in VE/VO_2_ without a concomitant increase in VE/VCO_2_ [22]. In a randomized, crossover design, the participants performed the protocol with the surges performed at three different intensities: at the intensity associated with their MAP, and at 25% and 50% of their APR. The intensity of the surges as a percentage of APR was calculated based on the formula below [18,21]:

25%APR PO = (APR x 0.25) + MAP

50%APR PO = (APR x 0.50) + MAP,

The participants were blinded to the intensity of the surges, but were notified 30 and 10 seconds prior to each surge, and a 5-second countdown was also provided. For each surge, the participants were asked to increase their cadence to a self-selected value above 110 rpm but were told to not perform these high-intensity efforts as an all-out effort. The participants were asked to complete every surge in the protocol, but were allowed to skip surges if they deemed it necessary to complete the protocol. When skipping a surge was needed, the participants stopped pedalling for the 10 seconds of the surge. The participants were not allowed to skip consecutive surges or to skip the final surge of the protocol. Once the last surge was performed, the participants completed 50 seconds of low intensity (60% MAP) cycling. Another 30 seconds of low intensity cycling (100W) were performed prior to the completion of a 30-second Wingate test. For the Wingate test, the participants were instructed to reach their maximal cycling cadence at the end of a 5-second audible countdown prior to the load being applied (7.5% of bodyweight) and to pedal all-out during the 30 seconds of the test. Strong verbal encouragement was provided during the test. An overview of the intermittent protocol can be seen in Fig 1.

**Fig 1:**
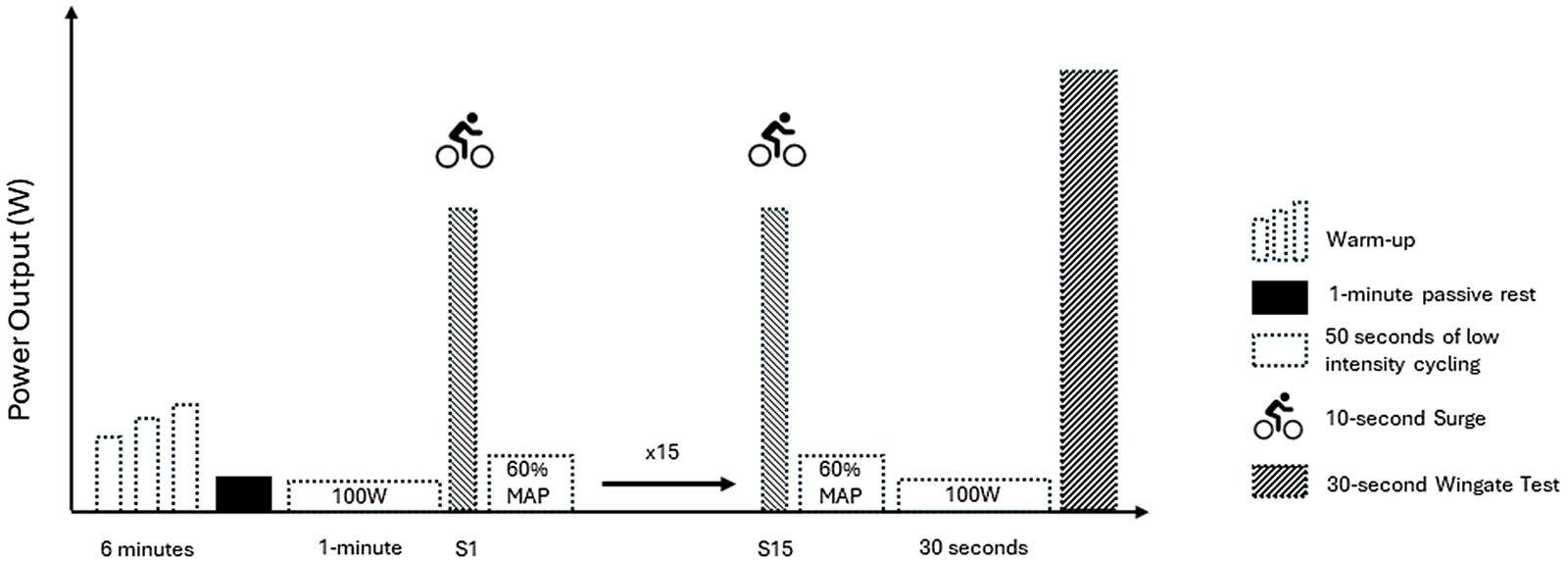
Overview of the intermittent endurance protocol. MAP: maximal aerobic power; S1: surge 1; S15: surge 15.

### Data collection

Data was collected immediately prior to the first 10-second surge and then after the 1^st^, 3^rd^, 6^th^, 9^th^, 12^th^, and 15^th^ surges, and during the 30-second Wingate test. Cardiorespiratory responses were collected as the average of a 30-second window that included the 10-second surge and the 20 seconds immediately after it. If a participant needed to skip a surge where data collection occurred, the data from the previous surge was included for analysis.

### Cardiorespiratory responses

For both the graded exercise test and high-intensity intermittent trials, the participants were connected to a gas analyzer (Power Lab, AD Instruments, Colorado Springs, CO, USA) to measure expired gases and determine oxygen consumption (VO_2_) and respiratory rate. The participant’s VO_2_max was determined as the highest 30-second value in the final 2 minutes of the graded exercise test. Throughout the tests, the participants also wore a chest strap (Garmin HRM Pro, Olathe, KS, USA) for continuous recording of heart rate.

### Blood lactate concentration

Blood lactate was measured via a finger prick using a portable blood lactate analyzer (Lactate Plus, Nova Biomedical, Waltham, MA, USA). Prior to the collection of the blood sample, the finger was cleaned using an alcohol swab and a cotton pad, and after the prick, the first drop of blood was discarded. Blood lactate was collected within 20 seconds of the participants completing a surge.

### Electromyographic activity

Electromyographic signals were recorded through a wireless electrode (Delsys Trigno Wireless, Boston, MA, USA). The myoelectric signal was recorded with a bandwidth frequency of 20–450 Hz and amplified (gain = x 1000). The data was collected using a sampling frequency of 2000Hz and extracted for analysis using the manufacturer’s software (Delsys EMG Works© and EMG Analysis©). EMG signals were processed offline using EMGworks Analysis (version 4.7.3.0; Delsys Inc.). A moving root-mean-square (RMS) envelope was calculated from the continuous EMG recording using a 3-second window with 50% overlap. The RMS envelope was overlaid on the raw EMG signal to inspect signal quality and confirm that the selected analysis epochs represented stable muscle activity without transient artefacts. The electrode was placed on the vastus lateralis of the right leg, with the placement following the recommendations of the SENIAM (Surface ElectroMyography for the Non-Invasive Assessment of Muscles) [23]. The area was cleared of body hair and cleaned using an alcohol swab and the sensors were placed as per manufacturer recommendations for adherence to the skin. The placement of the sensors was recorded using a plastic sheet and a permanent marker to ensure it was replicated on subsequent testing days. The EMG data was collected in 1.5-second windows and the average of the first 5 windows within each 10-second surge was utilized for analysis. During the Wingate test, the 5 windows represented the middle portion of the test (10–20 seconds). This window was chosen to ensure that there was no influence of the initial acceleration (up to 10 seconds) or from fatigue in the later portion (20–30 seconds) of the test.

Prior to the start of the high-intensity intermittent protocol on each testing day, the participants performed two 6-second Wingate tests [20]. The test was utilized as a measure of the participant’s maximal EMG activation during cycling [24]. This value was then used to normalize the EMG activity (EMG%) during the high-intensity intermittent protocol.

### Perceptual responses

Perceived fatigability was assessed within the trials through changes in the rating of perceived exertion (RPE) using the Borg 6–20 scale by asking the participants to rate their overall perceived exertion during the protocol [25]. The participants were also asked to assess their leg discomfort (heavy legs – HL), using a modified Borg CR-10 scale. The participants were asked “How heavy do your legs feel?” [25,26]. The scale’s anchor points ranged from “0 – nothing at all” to “10 – maximal” [26].

### Performance data

The maximal (MPP) and mean (MPO) power output during the 30-second Wingate were used to compare differences in performance between the conditions.

### Data analysis

Statistical analysis was performed using SPSS for Windows (SPSS 26.0, Chicago, IL). Data are presented as mean and standard deviation, with statistical significance set at p < 0.05. When applicable, 95% confidence intervals are also presented.

For the metabolic, perceptual, and neuromuscular data, a two-way repeated measures ANOVA (time vs condition) was used to assess interaction effects. Pairwise comparisons for simple main effects between the conditions at each time point were analyzed with a Bonferroni correction for multiple comparisons. Planned simple effects analysis using repeated contrasts to evaluate differences across adjacent time points within the same condition (i.e., pre vs S1, S1 vs S3, and so on) were performed, with the significance level adjusted to account for these multiple comparisons. Partial eta square (η^2^) effect sizes are presented and interpreted as small (η^2 =^ 0.01), medium (η^2^ = 0.06), and large (η^2^ = 0.14) [27].

If the assumption of sphericity was violated, a Greenhouse-Geisser correction was applied. Normality was tested using the Shapiro-Wilk test. Blood lactate data was log-transformed to meet the normality assumption, but this assumption was not met for perceptual measures (RPE and HL) and neuromuscular responses (EMG%). Given that ANOVA is robust to deviations from normality [28], these measures were still analyzed with a two-way repeated measures ANOVA to test for interaction effects. Changes across time points within each condition for these measures were analyzed using Friedman’s Test.

Maximal peak power (MPP) during the 30-second WG test was analyzed with Friedman’s test due to non-normal distribution, while mean power output values (MPO) were compared using one-way ANOVA.

## Results

The participants’ characteristics, graded exercise test results, maximal peak power (MPP), anaerobic power reserve (APR), and the intensity for each supramaximal condition are presented in Table 1. One participant skipped a surge during the MAP condition, while 7 and 11 participants skipped one or more surges in the 25APR and 50APR conditions, respectively. The physiological and perceptual responses throughout the protocol across the different conditions are presented in Fig 2.

**Table 1:** Participants’ characteristics.

Measure	Mean ± SD	95% Confidence intervals
Age (years)	36.2 ± 9.4	30.8–41.6
Bodyweight (kg)	84.9 ± 13.0	77.4–92.4
VO_2_max (L/min)	4.79 ± 0.80	4.34–5.23
VO_2_max (ml·kg^-1^·min^-1^)	57.6 ± 12.9	50.4–64.8
MAP (W)	333 ± 50	304–362
MPP (W)	1190 ± 186	1086–1293
APR (W)	836 ± 189	731–940
25% APR (W)	542 ± 72	501–582
25% APR as a percentage of MAP (%)	164 ± 16	155–172
50% APR (W)	751 ± 110	690–812
50% APR as a percentage of MAP (%)	228 ± 32	210–246

**Fig 2:**
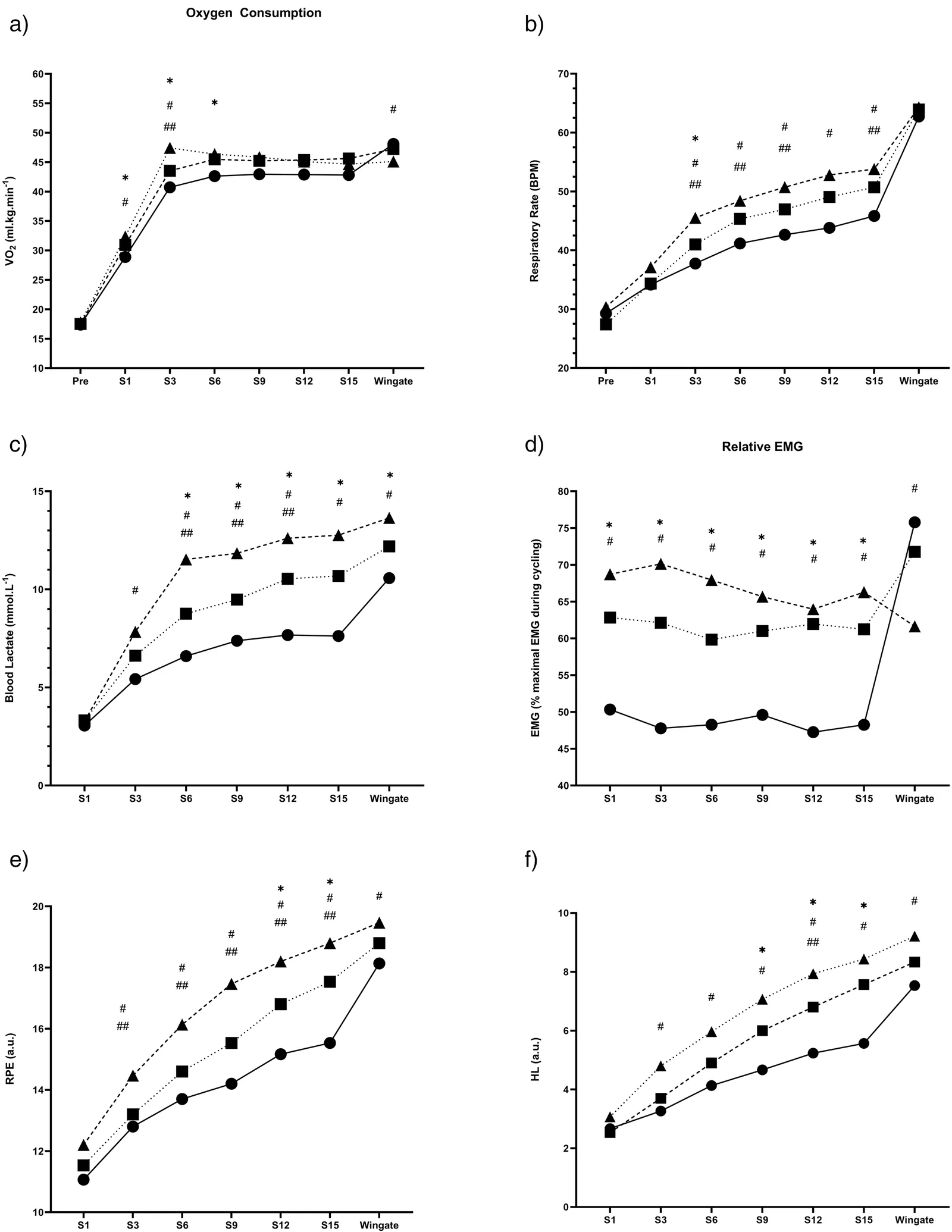
Physiological responses throughout the protocol across the different conditions. a) oxygen consumption (VO_2_), b) respiratory rate, c) blood lactate, d) EMG as a percentage of maximal EMG activation during cycling, e) rate of perceived exertion (RPE), f) heavy legs (HL). MAP: maximal aerobic power (circles), 25APR: 25% of the participant’s anaerobic power reserve (squares), 50APR: 50% of the participant’s anaerobic power reserve (triangles) ^*^ MAP significantly different than 25APR, ^#^ MAP significantly different than 50APR, ^##^ 25APR significantly different than 50APR. Please note the Y-axis for Fig 2e (RPE) has been truncated. Error bars have been omitted for clarity and can be seen for each condition in the supplementary files (Figs 4–6 in S1 File).

### Physiological and perceptual responses

The interaction effect was significant for VO_2_ (F(4.29, 60.19) = 3.71, p = .008, partial η^2^ = .209), respiratory rate (F(4.98, 69.81) = 3.86, p = .004, partial η^2^ = .216), blood lactate (F(4.10, 57.51) = 4.72, p = .002, partial η^2^ = .252), EMG% (F(4.67, 65.45) = 13.99, p < .001, partial η^2^ = .500), RPE (F(5.79, 81.08) = 4.64, p < .001, partial η^2^ = .249) and for HL (F(4.64, 64.99) = 6.31, p < .001, partial η^2^ = .311) (Fig 2a to 2f).

The main effect of condition was significant for heart rate (F(2, 28) = 7.27, p = .003, partial η^2^ = .342), with higher values for MAP compared to the 50APR condition (p = .007); blood lactate (F(2, 28) = 24.42, p < .001, partial η2 = .636), with lower lactate levels in MAP vs 25APR (p = .002), MAP vs 50APR (p < .001), and in 25APR vs 50APR (p = .021); respiratory rate (F(2, 28) = 18.12, p < .001, partial η^2^ = .564), with higher values for 50APR compared to MAP (p < .001) and to 25APR (p = .005); EMG% (F(2, 28) = 9.37, p < .001, partial η^2^ = .401), with lower EMG% in MAP compared to 25APR (p = .013) and MAP compared to 50APR (p = .014); RPE (F(1.43, 20.08) = 15.20, p < .001, partial η^2^ = .521), with significantly lower RPE for MAP compared to 25APR (p = .045) and to 50APR (p = .002), and lower values for 25APR compared to 50APR (p = .004); and HL (F(2, 28) = 15.56, p < .001, partial η^2^ = .526), with lower values in MAP compared to 25APR (p = .020) and to 50APR (p = .001), and in the 25APR condition compared to 50APR (p = .022).

In each condition, the main effect of time was significant (p < .001) for all physiological and perceptual measures (Figs 4–6, S1 File).

### Performance data

There was a significant main effect of condition for MPP (χ^2^ = 10.53, p = .005), with a significant difference (p = .006) between MAP (median = 1036W, interquartile range [942W – 1223W]) and 50APR (median = 946W, interquartile range [899W – 1054W]) (Fig 3a).

**Fig 3:**
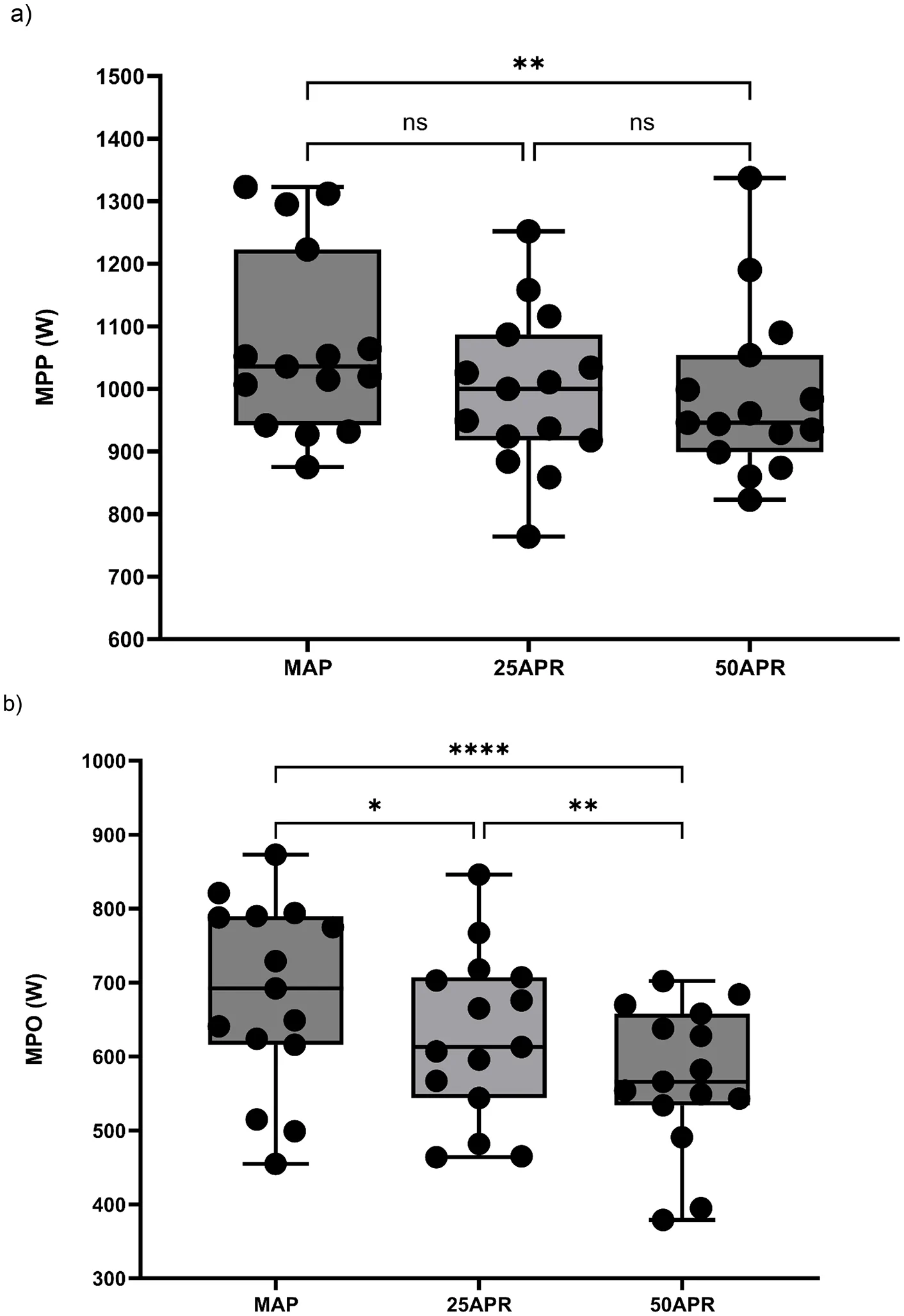
Performance during the 30-second Wingate test. a) Maximal peak power (MPP), b) mean power output (MPO); MAP: maximal aerobic power, 25APR: 25% of the participant’s anaerobic power reserve, 50APR: 50% of the participant’s anaerobic power reserve; ns: non-significant, *p < 0.05, **p < 0.01, ****p < 0.001.

For MPO, a significant difference between the conditions was reported (F(2, 28) = 29.11, p < .001, eta square = .675). MPO was significantly different between MAP and 25APR (p = .010, mean difference = 56.0W, 95% CI = 13 to 99W), MAP and 50APR (p < .001, mean difference = 112W, 95% CI = 74 to 151W), and 25APR and 50APR (p = .004, mean difference = 56W, 95% CI = 18 to 95W) (Fig 3b).

## Discussion

The results showed that both APR conditions led to a fast rise in physiological and perceptual responses, reaching very high or limiting values within the first 6 surges (total of 60 seconds of total intense work performed), with these responses being intensity dependent (i.e., greater demands in 50APR compared to MAP and 25APR, and in 25APR compared to MAP). The ability to complete repeated bouts at 25% and 50% of the athlete’s APR was limited, with only 8 and 4 participants (out of 15) completing all 15 surges in each condition, respectively. Performance in the 30-second Wingate was significantly impaired, proportional to the intensity of the high-intensity intermittent protocol.

### Physiological responses to intermittent endurance exercise within the anaerobic power reserve

Few differences were seen in aerobic metabolism between the conditions, despite the higher demands of performing surges at 25APR and 50APR. The APR conditions led to a greater VO_2_ demand within the first 3 surges (Fig 2a). This rapid spike at the onset of the protocol (S1) is characteristic of the exponential increase in VO_2_ when exercise is performed at these intensities [29]. The subsequent higher values seen in the APR conditions (at S3 for 25APR and 50APR, and S6 for 50APR) likely indicates an increase in oxidative metabolism to support energy production for further surges [30,31] as well as the rise in O_2_ demand during the recovery processes following intense efforts (e.g., PCr resynthesis) [32,33]. Heart rate responses have a similar pattern to that of VO_2_, with higher values early in the protocol being responsible for the higher overall mean of the 50APR condition compared to MAP. The similar responses across conditions throughout the remainder of the protocol are likely due to the short duration of the surges and the recovery period between efforts, with HR and VO_2_ attaining levels consistent with previously reported findings in intermittent endurance exercise at similar intensities (approximately 80–85% and 90–94% of their maximal values, respectively) [2,17,34].

The responses to each condition also indicate that the APR conditions likely required a greater anaerobic contribution to support their increased energy demands. This is supported by elevated EMG activity (approximately 60% and 70% of the participant’s maximal EMG activation during cycling for the 25APR and 50APR, respectively) (Fig 2d), likely indicating increased recruitment of type II glycolytic muscle fibers. While the similar blood lactate concentrations for each condition following S1 suggest that phosphocreatine (PCr) stores served as the primary energy source for this effort [30,35], incomplete PCr replenishment during the 50-second recovery [11,36,37] likely accelerated glycolytic flux [33]. As such, blood lactate concentrations increased rapidly in the 25APR and 50APR conditions (approx. 8–10 mmol.L^-1^ as early as S6 in each condition, respectively) (Fig 2c), values that are akin to what has been reported at task failure during an intermittent work within the APR (albeit in running) [17,18]. In contrast, the MAP condition only reached comparable values (e.g., 10.5 mmol.L^-1^) post-Wingate. The quick attainment of such high values of blood lactate in the APR conditions highlights that the periods of low intensity were insufficient to attenuate the metabolic demands of intermittent work at these intensities.

The increased reliance on glycolysis elevates H^+^ concentrations and potentially leads to a reduction in pH within the muscle [14], both factors associated with impaired muscle contractile capacity [13]. These two factors also contribute to an increase in respiratory rate, which is driven by the increase in CO_2_ (a by product of the buffering reactions needed to offset the increase in H^+^) and by a decline in blood pH [38]. Indeed, blood lactate and respiratory rate showed a similar pattern of response between the conditions, with significantly higher respiratory rate values in the 25APR and 50APR conditions compared to the MAP condition as early as S3 (Fig 2b). The elevated respiratory rate also leads to an increased work of breathing secondary to an increase in dead-space ventilation, which can divert blood flow from the locomotor muscles to the respiratory muscles, contributing to the rapid increases in feelings of fatigue [39].

Perception of effort (RPE) (Fig 2e) and limb discomfort (HL) mirrored these physiological responses, with RPE in particular, displaying a similar pattern to respiratory rate. The RPE during exercise also reflects the degree of disturbances to numerous systems (e.g., skeletal muscles, cardiorespiratory system), the sensation of how strenuous a task feels, and is considered a major feature of fatigue [26,40,41]. The integration of these responses likely explains the significantly higher RPE values attained earlier in the protocol, particularly for the 50APR condition. Limb discomfort (heavy legs) [26] also showed higher values in the APR conditions likely due to the greater degree of localized muscle metabolic perturbations previously discussed. The gradual increase in both measures throughout the protocol in the supramaximal conditions (particularly in the 50APR condition) is an important observation as sense of effort plays a key role in how participants regulate their power output during repeated sprint efforts [26].

### Is intermittent endurance exercise within the anaerobic power reserve sustainable?

Contrary to our hypothesis, the participants decided to skip surges to ensure completion of the protocol at these intensities, with only 8 and 4 participants (out of 15) completing all surges in the 25APR and 50APR conditions, respectively. The work performed at these intensities led to a severe level of metabolic stress within the muscle, as previously discussed, with the recovery period being insufficient to minimize the metabolic disturbances associated with the efforts. As such, perceived fatigability significantly increased, with limb discomfort (HL) rated as severe to very severe by S6 in the APR conditions and RPE rated as “very hard” as early as S6 (15 and 16 out of 20, for 25APR and 50APR, respectively). As athletes manage their efforts based on RPE, along with their knowledge of the distance remaining in the race [40,42,43], the high RPE values early in the protocol probably led the participants to perceive the intermittent work as unsustainable without a reduction in intensity. While surges in intensity at higher percentages of APR (e.g., 50APR) are less frequent in endurance events, the difference in responses (and exercise tolerance) between the 25APR and 50APR conditions emphasize the importance of minimizing the percentage of APR at which these efforts occur during a race.

The participants’ decision to skip sprints contrasts with other studies in the literature [9,44] in which participants were able to perform multiple work intervals at similar or all-out intensities. However, it is likely that differences in the duration of the efforts (6-second vs 10-second sprints) [44], or in the recovery duration and intensity (50 seconds at 60% MAP vs 60 seconds at 45% MAP) [9] account for the different results. Further, differences in APR values between the studies might also have played a role, as individuals with a higher APR (as was the case in this study) likely rely more on anaerobic energy to sustain these efforts, which can contribute to a larger degree of metabolic stress [8,44] and the aforementioned elevated RPE, contributing to the participants’ perception that skipping a sprint was necessary to cope with the task.

### Performance during a 30-second Wingate following intermittent exercise

The different intensity of the surges in each condition also led to differences in the total work done (TWD) during the protocol, with the TWD for the MAP condition representing only 66% and 55% of the work performed in the 25APR and 50APR conditions, respectively. As such, the significant differences in mean power output (MPO) (684W vs 628W vs 571W, for MAP, 25APR, and 50APR, respectively) (Fig 3b) were expected, and these results should be analyzed with caution. Nevertheless, previous studies have shown that intensity plays a role in subsequent performance, particularly in short efforts (less than 3 minutes) when total work done is equated between conditions [15,45]. Thus, the intensity of the efforts likely had an influence on performance, despite the differences in total work done.

Maximal peak power (MPP), however, was only significantly different between the MAP and 50APR conditions (1071W vs 988W, respectively) (Fig 3a). These results corroborate the assertion that instantaneous power is not impacted to the same extent as the ability to perform maximal efforts of longer durations (15–30 seconds) [15]. It is also possible that the longer recovery (about 90 seconds) between the end of the high intensity intermittent protocol and the start of the Wingate test allowed sufficient time for PCr replenishment to occur, minimizing the differences in performance between the conditions. While an important finding, the finishing sprint within an endurance race can last up to 20–25 seconds [6]. As such, the ability to sustain a high-power output for the duration of the effort is more important than generating a high peak power at its onset.

Given the importance of the finishing sprint to overall performance in endurance events, our results show that the athlete that can stay with the race leaders while minimizing the demands of the race prior to the finishing sprint (i.e., performing efforts at a lower percentage of APR) is more likely to produce a successful effort in this race-defining moment.

### Limitations

A key aspect of this study was allowing participants to skip surges to ensure completion of the protocol. This reflects a more realistic scenario, as athletes are often “dropped out” during a race when they can no longer remain with the pack. Nevertheless, this also leads to limitations related to interpretation of our data. First, when a participant decided to skip a surge, data from the surge performed immediately before was utilized. Given that fatigue at such high intensities (25APR and 50APR) might increase exponentially between efforts [10], it is possible that this led to an underestimation of the values reported. Further, allowing the participants to skip surges might have introduced a degree of variability in their responses to the protocols. The participants that skipped a surge likely did so to ensure physiological and perceptual responses remained within values that they were able to cope with. As such, it is possible that the lack of differences in some responses between the conditions in later time points (e.g., VO_2_ between S9 – S15) is due to a reduction in these values during the longer recovery period when a participant chose to skip a surge. Nevertheless, our data shows a similar pattern of change in physiological and perceptual responses as other studies in the literature, where a rapid increase in these responses occurs followed by a plateau at very high to near maximal levels. A further limitation to be acknowledged is related to our data collection. Physiological data (e.g., VO_2_, respiratory rate) was collected in 30-second windows (including the 10-second surge and the subsequent 20 seconds) which may have attenuated the values presented. Nevertheless, our results were still able to show significant differences between the conditions early in the protocol (e.g., surges 1–6), with the lack of differences later in the protocol (e.g., VO_2_) likely more related to the participants skipping sprints than to the window utilized for analysis.

Despite these limitations, our results might be reflective of what occurs in real-race situations, where athletes must decide if they are willing to engage in high-intensity work with the group, or stay behind and potentially lose contact with the pack. As such, it also provides an interesting discussion point related to the physiological and perceptual responses when participants decided to skip a surge. For example, when a participant skipped a surge, the decision was made based on their responses at the time point for which data is presented. Therefore, we are confident that the data not only represents the highest values that the participants were willing to cope with to ensure completion of the protocol, but it can also inform how athletes should manage the demands of a race to ensure optimal performance in a similar situation.

## Conclusion

The results in this study show that intermittent endurance exercise (10-second surges with 50 seconds of moderate intensity work) performed within the APR leads to a fast attainment of high blood lactate levels and significantly elevated respiratory rate that could imply a limiting intramuscular environment (i.e., metabolic acidosis). Concomitantly, perceptual responses (e.g., RPE) are exacerbated. Exercise at these intensities was not universally sustainable, with approximately 50% and 75% of the participants in the 25APR and 50APR conditions deciding to skip surges to complete the protocol. In contrast, only 1 participant skipped a surge in the MAP condition.

Athletes involved in endurance events that require supramaximal efforts should consider the consequences of performing surges at these intensities. Training to increase MAP can allow athletes to sustain a higher work intensity prior to transitioning into supramaximal intensities. Further studies should also investigate if raising the upper limit of the athlete’s APR by training to increase the athlete’s MPP (either via resistance training or by performing short, all-out efforts) is an effective way to minimize the percentage of the APR that is utilized during supramaximal efforts. Both strategies can minimize the metabolic disturbances associated with these efforts, reducing the reliance on anaerobic energy, and improving the athlete’s ability to complete further work in the race, leading to better performance in the finishing sprint. Increasing an athlete’s ability to withstand the demands of performing surges at a higher intensity might lead to a tactical advantage over the competition, allowing athletes to establish breakaways at a higher work rate (or for a longer duration) to force others into unsustainable intensities.

## Supporting information

S1 File Figures 4–6. Physiological responses throughout the protocol during the MAP condition (figure 4), 25APR condition (figure 5), and 50APR condition (figure 6). a) oxygen consumption (VO_2_), b) respiratory rate (RR), c) blood lactate, d) EMG as a percentage of maximal EMG activity during cycling (%EMG), e) rate of perceived exertion (RPE), f) heavy legs (HL). ^*^ significantly different from previous time point (p < .05), ^#^ significantly different from previous time point (p < .01).
(DOCX)
