# Supplementary material for: The physiological demands of intermittent endurance exercise performed at different intensities within the anaerobic power reserve: Implications for fatigue development and performance
Source: PLoS One. 2026 Nov 13;21(11):e0359318. doi: 10.1371/journal.pone.0359318 (PMC13766254; doi:10.1371/journal.pone.0359318)
Supplement: Supplementary file 1 [file pone.0359318.s001.docx]

| a)   | b)   | |
| --- | --- | --- |
| c)   | d)   | |
| e)   | f)   |  |

**Figure 4.** Physiological responses throughout the protocol during the MAP condition. a) oxygen consumption (VO_2_), b) respiratory rate (RR), c) blood lactate, d) EMG as a percentage of maximal EMG activity during cycling (%EMG), e) rate of perceived exertion (RPE), f) heavy legs (HL). ^*^ significantly different from previous time point (p < .05), ^#^ significantly different from previous time point (p < .01).

| a)   | b)   | |
| --- | --- | --- |
| c)   | d)   | |
| e)   | f)   |  |

**Figure 5.** Physiological responses throughout the protocol during the 25APR condition. a) oxygen consumption (VO_2_), b) respiratory rate (RR), c) blood lactate, d) EMG as a percentage of maximal EMG activity during cycling, e) rate of perceived exertion (RPE), f) heavy legs (HL). ^*^ significantly different from previous time point (p < .05), ^#^ significantly different from previous time point (p < .01).

| a)   | b)   | |
| --- | --- | --- |
| c)   | d)   | |
| e)   | f)   |  |

**Figure 6.** Physiological responses throughout the protocol during the 50APR condition. a) oxygen consumption (VO_2_), b) respiratory rate (RR), c) blood lactate, d) EMG as a percentage of maximal EMG activity during cycling, e) rate of perceived exertion (RPE), f) heavy legs (HL). ^*^ significantly different from previous time point (p < .05), ^#^ significantly different from previous time point (p < .01).
